# Supplementary material for: Content-rich biological network constructed by mining PubMed abstracts
Source: BMC Bioinformatics. 2004 Oct 8;5:147. doi: 10.1186/1471-2105-5-147 (PMC528731; doi:10.1186/1471-2105-5-147)
Supplement: Additional File 5 — The original Chilibot query results of the term "long-term potentiation (LTP)" and 22 other terms, limiting the latest references analyzed to the years 1990, 1995, 2000, and 2004. [file 1471-2105-5-147-S5.bz2 › chilibotAdditionalFile5/ltp2000/html/SYNAPSIN I_SYNAPTOTAGMIN.html]

 


 **SYNAPSIN I** and **SYNAPTOTAGMIN** 
  
Found 17 abstracts in PubMed,  **17 abstracts were retrieved and analyzed**.  


---

 Search Google  |
 PDF files only 
|  EDU domain only 

---

**Interactive relationship** (e.g. stimulation, inhibition, etc)

**Parallel relationship** (e.g. studied together, co-existance, homology, etc.)

- At both 4 and 12 DIC, the synaptic proteins rab3a,  **synapsin I** , and  **synaptotagmin**  were present in hippocampal neurons, but the subcellular distribution changed from one in which immunoreactivity was initially distributed within soma and neurites to a punctate varicose appearance.  Ref: 7965045 J Neurosci, 1994
- To investigate the changes induced in presynaptic protein phosphorylation by 5 HT reuptake blockade and concomitant CaMKII up regulation, we analyzed two major CaMKII presynaptic substrates  **synapsin I**  and  **synaptotagmin** .  Ref: 9016342 Mol Pharmacol, 1997
- VAChT containing varicosities in the Renshaw cell area, with a probable origin from motoneuron axon collaterals, exhibited CSP,  **synapsin I**  II, and synaptobrevin 1 LI... syntaxin, synuclein,  **synapsin I** ,  **synaptotagmin**  I,  **synaptotagmin**  I II, synaptophysin and synaptobrevin 2 LI.  Ref: 10421869 J Comp Neurol, 1999
- Immunohistochemistry in combination with confocal laser microscopy showed the presence of synaptosomal associated protein of 25 kDa, syntaxin, cysteine string protein, synuclein,  **synapsin I** ,  **synapsin I**  II,  **synaptotagmin**  I,  **synaptotagmin**  I II, synaptophysin, and synaptobrevin 2 like immunoreactivity in VAChT containing C terminals.  Ref: 10421869 J Comp Neurol, 1999
- Horseradish peroxidase filled vacuoles as wells as vesicles contained the synaptic vesicle membrane proteins VAMP II,  **synaptotagmin** , SV2, and synaptophysin, the vesicle associated proteins rab 3A and  **synapsin I** , and in addition SNAP 25.  Ref: 10579591 Neuroscience, 1999
- The effect of LTX on phosphorylation of synaptosomal proteins was studied including  **synapsin I** ,  **synaptotagmin** , and dynamin.  Ref: 8998284 Biokhimiia, 1996
- Despite the nearly constant rates of synthesis of  **synaptotagmin**  I, vamp 2, and  **synapsin I** , we show that the number of SVs in these developing neurons increases,.  Ref: 9065497 J Neurosci, 1997
- The conditions used indicate that  **synaptotagmin** , as  **synapsin I** , is phosphorylated by calcium calmodulin dependent protein kinase II.  Ref: 8381369 FEBS Lett, 1993
- The presence of synaptic vesicle associated proteins such as synaptophysin,  **synaptotagmin**  and  **synapsin I**  in the growth cones of regenerating axons indicates the possibility that exocytotic fusion of vesicles with the surface axolemma supplies the membranous components for the extension of regenerating axons.  Ref: 8829147 Neurosci Res, 1996
- Rab3a and synaptobrevin II were retained at a high level and  **synaptotagmin**  I,  **synapsin I** , and syntaxin I at a lower level until 20 DIV.  Ref: 10340764 Glia, 1999
- High magnification revealed that  **synaptotagmin**  I like immunoreactivity was mainly distributed in a fine granular pattern, but large, brightly fluorescent granules which were not labelled by anti synaptophysin or anti  **synapsin I**  were occasionally observed.  Ref: 7534885 Neuroscience, 1994
- No  **synaptotagmin**  I like immunoreactivity was found in large axons, while accumulations of the synaptic vesicle proteins synaptophysin and  **synapsin I**  were found in all types of axons.  Ref: 7534885 Neuroscience, 1994
- **Synaptotagmin**  I, vamp 2, and  **synapsin I**  proteins are synthesized at nearly constant rates as the neurons develop.  Ref: 9065497 J Neurosci, 1997
- We measured the levels of seven presynaptic proteins synaptobrevin,  **synaptotagmin** , SNAP 25, syntaxin, SV2, Rab3a, and  **synapsin I**  by immunoblotting in postmortem tissue from four brain regions hippocampus, entorhinal cortex, caudate nucleus, and occipital cortex.  Ref: 10831767 J Neurol Sci, 2000
